# Supplementary material for: Splice-Junction-Based Mapping of Alternative Isoforms in the Human Proteome
Source: Cell Rep. Author manuscript; Available in PMC 2020 Jan 15. (PMC6961840; doi:10.1016/j.celrep.2019.11.026)
Supplement: 2 [file NIHMS1546469-supplement-2.zip › DF1/PXD010154/Thyroid/Thyroid_27_TG_NSDMGSRPESMGCR.pdf]

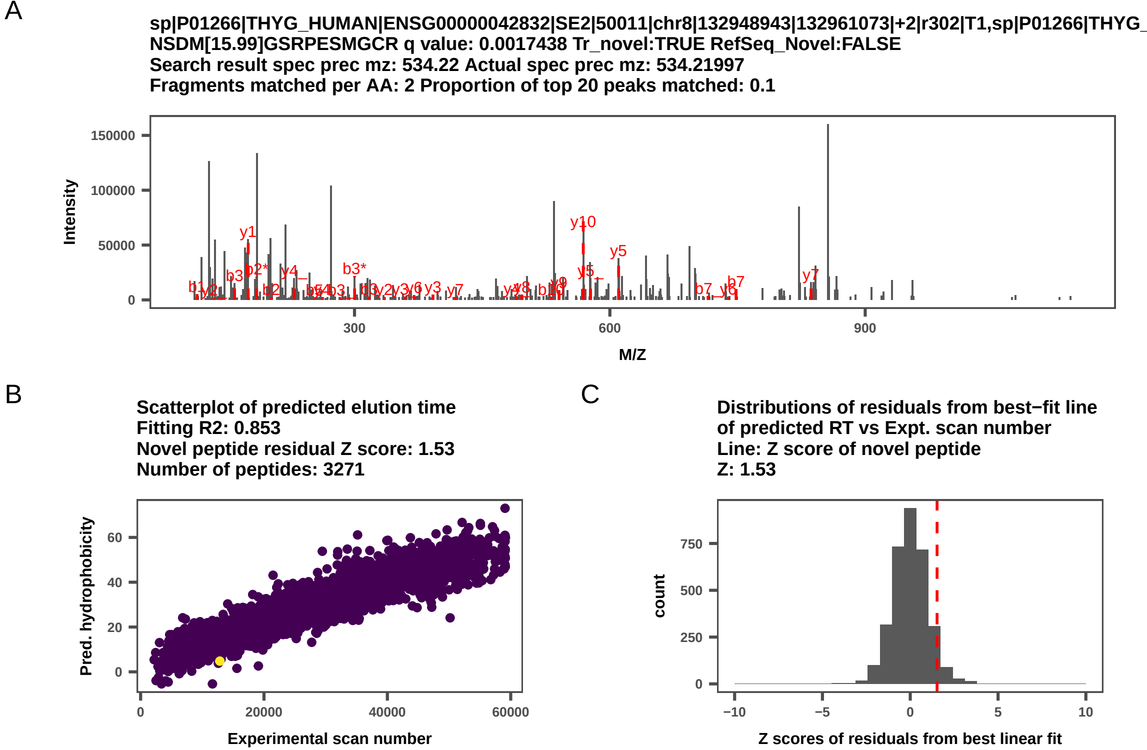
A
sp|P01266|THYG_HUMAN|ENSG00000042832|SE2|50011|chr8|132948943|132961073|+2|r302|T1,sp|P01266|THYG_
NSDM[15.99]GSRPESMGCR q value: 0.0017438 Tr_novel:TRUE RefSeq_Novel:FALSE
Search result spec prec mz: 534.22 Actual spec prec mz: 534.21997
Fragments matched per AA: 2 Proportion of top 20 peaks matched: 0.1
Intensity
150000
100000
50000
0
300
600
900
M/Z
y1
y10
y5
b3*
b2*
y4
y7
b7
B
Scatterplot of predicted elution time
Fitting R2: 0.853
Novel peptide residual Z score: 1.53
Number of peptides: 3271
Pred. hydrophobicity
60
40
20
0
0
20000
40000
60000
Experimental scan number
C
Distributions of residuals from best−fit line
of predicted RT vs Expt. scan number
Line: Z score of novel peptide
Z: 1.53
count
750
500
250
0
−10
−5
0
5
10
Z scores of residuals from best linear fit
